# Toileting and Bladder Health in the Gig Economy

**DOI:** 10.1002/nau.70122

**Published:** 2025-07-27

**Authors:** Leah Chisholm, Andria N. Li, Parisa Samimi, Melissa R. Kaufman, Roger R. Dmochowski, William Stuart Reynolds, Elisabeth M. Sebesta

**Affiliations:** ^1^ Department of Urology Vanderbilt University Medical Center Nashville TN USA; ^2^ Department of Urology University of Michigan Ann Arbor MI USA

**Keywords:** bladder health, toileting behavior, workforce

## Abstract

**Objective:**

An increasing percentage of the population participates in the “gig economy” – short‐term work through online applications. This study often requires continuous travel without reliable restroom access. We aimed to assess toileting behaviors and bladder health in gig economy workers.

**Methods:**

Adult gig workers were electronically recruited using ResearchMatch. Participants completed validated questionnaires assessing bladder health, lower urinary tract symptoms (LUTS), toileting behaviors, and information about gig economy work. Toileting behaviors, coping strategies, and aspects of their work were compared between those with and without difficulty finding restrooms while at work.

**Results:**

Of 527 gig workers who participated, 80 (15%) reported difficulty finding restrooms while working. Demographics and type of gig work were similar between groups. Gig workers with difficulty finding restrooms reported higher rates of unhealthy toileting behaviors and coping strategies, such as fluid restricting and delayed voiding. While at work, those with difficulty finding restrooms experienced more severe LUTS. While difficulty finding restrooms was not associated with any psychosocial or demographic factors, it was associated with worsening of urinary symptoms since starting gig work.

**Conclusions:**

Gig workers overall report engaging in unhealthy toileting behaviors and coping strategies at work. Gig workers with difficulties in accessing restrooms while working report higher severity of irritative LUTS and are more likely to demonstrate poor toileting habits, which may further exacerbate underlying bladder conditions. Further research is needed to better understand the long‐term associations between bladder health, restroom access and employment in the gig economy.

**Clinical Trial Registration:** This study does not require clinical trial registration as no randomized clinic trial was performed.

## 1 Introduction

Lower urinary tract symptoms (LUTS) are highly prevalent in the United States (U.S.), and are associated with unhealthy bladder habits, such as delayed voiding and limiting restroom use, and overall poor bladder health [1, 2, 3, 4, 5]. The associations between toileting behaviors, LUTS, and overall bladder health are complex. Furthering our understanding of how social and environmental factors, such as those related to occupation, impact the development of LUTS continues to be important, as relatively few studies to date have examined LUTS across different occupations [6].

Previous literature on the development of LUTS and the workplace has suggested that infrequent voiding, limited toilet access, and other restrictions to using the bathroom at work may contribute to the development of LUTS in certain occupations. Likewise, various occupations where delayed and infrequent voiding are common have been linked to poor bladder health. For instance, health care professionals, who often delay voiding while at work, report increased rates of urinary incontinence and overactive bladder (i.e. “nurse's bladder”) [7, 8, 9, 10, 11]. A similar pattern has been described in New York City taxi drivers, (i.e. “taxi cab syndrome”) where infrequent voiding among professional taxi cab drivers is associated with increased incidence of voiding dysfunction [12]. In these professions infrequent voiding is often related to limitations in restroom access, due to lack of available restrooms or job‐related burdens impeding restroom use (i.e. time‐pressure demands, lack of or specified time and duration of bathroom breaks, “patient first” framework of health care resulting in self‐imposed restrictions on personal time including breaks, etc.) [13]. Most of the existing literature on toileting and LUTS as it relates to occupation focuses on the traditional workplace, a public space away from home. However, as short‐term work, including the gig economy, grows, lack of restrooms on the job poses a challenge for workers in this space.

The “gig economy” refers to the use of digital platforms to connect workers with short‐term, freelance, on‐demand jobs. In the U.S., it is estimated that up to 30% of the working‐age population is involved in independent work, with approximately 15% using an “online platform.” [14] The numbers of workers in the gig economy will likely continue to grow and age, as older workers enter into the workforce [15]. However, little is known about the health‐specific demands of the gig economy. In general, limited protections are available for workers who are commonly classified as “independent contractors.” Thus a gig worker is often uninsured through their gig position, has increased exposure to occupational social and environmental hazards, and experiences job instability [16].

The unknown health demands of gig work pose many questions regarding potential healthcare outcomes, including bladder health. Like taxi drivers, gig work typically requires travel, and the lack of physical work site for workers presents limited opportunities for toilet access and use. Therefore, gig workers may be at risk for developing and using maladaptive toileting habits, which in turn may impact bladder health and result in new or exacerbated LUTS. Alternatively, with some increased flexibility associated with gig work as compared to taxi driving, there could be a protective effect to reduce LUTS. As there have been no studies to date evaluating bladder health among gig workers, the goal of this study was to determine whether there are associations between gig work and bladder conditions and LUTS. We hypothesized that gig workers who report difficulty finding restrooms while at their job would describe maladaptive toileting habits and more frequent and severe LUTS. To test this hypothesis, we recruited a convenience sample of self‐identified, community‐dwelling gig economy workers and assessed for differences in LUTS, bladder health, compensatory toileting behaviors, and toilet work environments.

## 2 Methods

### 2.1 Subject Recruitment and Sample

After Institutional Review Board approval, we recruited adult men and women registered with ResearchMatch to participate in this cross‐sectional study via email solicitation. ResearchMatch is a national health volunteer registry of over 145,000 people that was created by several academic institutions and supported by the U.S. National Institutes of Health as part of the Clinical Translational Science Award (CTSA) program [17, 18]. The CHERRIES checklist was used in both the creation of the survey and analysis of the results [19]. Between January and March 2020, potential subjects received a one‐time email notification to participate in the study and were incentivized to complete the study with a random chance to receive one of ten $100 gift cards. Consenting participants completed an English‐only, anonymous electronic questionnaire administered through REDCap. 780 (0.5%) interacted with the email and started the questionnaire.

We included any respondent who indicated they participated in the gig economy as their primary or secondary employment. From the one‐time study notification, we recruited 780 subjects. Participants were excluded if they were currently pregnant, younger than 18 years old, they reported using a catheter to manage their bladder, had a history of neurogenic bladder dysfunction or did not fully complete the questionnaire. After applying the exclusion criteria, 527 participants were included in the final study analytic sample, which is 67.6% of those who interacted with the recruitment email. No data was available from non‐responders.

### 2.2 Study Measures

Questionnaire items collected self‐reported information on participant demographics, gig work specifics, medical history, LUTS, and toilet and bladder behaviors. Subjects provided information on age, race/ethnicity (White, Black, Asian, Hispanic, Other/Native American/Multiracial), gender (male, females, nonbinary), height and weight (for body mass index [BMI] calculation), highest education (high school or less, college or more), residence locality (urban, suburban, rural), and health insurance status (uninsured, self‐insured, government insured, insured through non‐gig employer, insured through gig employer, insured through partner). Per previously proposed bladder health life stages, we categorized patient based on the following age group ranges: 18–24 years, 25–44 years, 45–74 years and ≥ 65 years [1].

Bladder health and LUTS were assessed with several questionnaires. Using the International Consultation on Incontinence LUTS Questionnaire (ICIQ‐LUTS) with a 4‐week recall, we calculated a total LUTS score and subscales for filling, incontinence, voiding and overactive bladder using published methods [20]. Items from both ICIQ‐FLUTS and ICIQ‐MLUTS were also incorporated in the questionnaire to accommodate both male and female participants in our study. We also defined individual LUT conditions based on whether the participant selected at least “sometimes” for urgency, urgency urinary incontinence (UI), stress UI and mixed UI (defined as urgency UI and stress UI). We defined OAB as urgency and/or urgency UI at least sometimes [21]. We assessed UI with the ICIQ‐Urinary Incontinence Short Form (ICIQ‐UI SF) as a validated measure of frequency and severity of UI among males and females within a 4‐week recall [22]. The Patient Perception of Bladder Condition (PPBC) was used to assess participant self‐perception of their bladder health. Subjects were dichotomized into 2 groups based on responses [23] (i.e. “none” or “very minor problems” vs. at least “some minor problems”) [5]. Subjects reported whether they had recurrent urinary tract infections (UTIs) (defined as 3 or more UTIs in the past year), prior kidney stones or diagnosis of interstitial cystitis/bladder pain syndrome. To assess how LUTS may have changed during their gig work, we administered a modified version of the Patient Global Impression of Improvement (PGI‐I) [24] “Compared to before you started working in your gig, please describe how your bladder symptoms are now” with 7‐point Likert responses, ranging from “very much worse” to “very much better.”

We administered several questions from the Toileting Behaviors: Women's Elimination Behaviors (TB‐WEB) scale to assess bladder toileting behaviors, focusing on domains of place preference, premature voiding, delayed voiding, straining to void, and position preference [25]. Individual toileting behaviors were defined by whether or not a worker performs the behavior at least often (i.e. often or always vs. never, occasionally, or sometimes), representing habitual behavior [4].

Regarding gig work characteristics, we asked participants to report type of gig work (ride sharing platform, food delivery, elder and childcare, pet care, technology testing, skilled labor platform, service‐based platform, home care, other), hours per week at gig job, and duration in months of employment within gig job. We assessed additional bladder coping strategies while at work, including limiting restroom use, restricting fluid intake, bringing extra clothes in case of UI, changing clothes for UI, or using UI products.

To assess use and perceptions of public restrooms, participants reported whether they limited public restroom use by answering “Do you purposefully limit your use of the restroom while you are working?” (not at all, occasionally, sometimes, most of the time, all of the time). Those responding at least occasionally were also asked to select reasons why (“quality of the restroom is poor”, “limited availability of a bathroom”, being busy”, “embarrassment or privacy”, “no need”, “lack of gender options”, “other reason”).

### 2.3 Statistical Analyses

Our primary exposure measure was whether gig economy workers reported difficulty finding a restroom while at their job, categorized as either never/rarely/sometimes or often/always to reflect habitual behavior. Primary outcome measures included the variables described above. We summarized data with mean and standard deviation (SD, continuous variables) or frequency and percentage (categorical variables) and compared differences between our primary exposure using t‐tests or chi‐squared tests, as appropriate. To evaluate the association between our exposure (i.e. whether an individual reported difficulty finding a restroom) and whether their urinary symptoms changed while at their gig job, we fitted a multivariable logistic regression model, adjusting for participants’ age, gender, BMI, months at gig job, educational level, and symptom bother (PPBC). Odds ratios (ORs) and associated 95% confidence intervals (CI) were reported as effect measurements. Statistical significance was considered for all two‐sided *p* values < 0.05.

## 3 Results

A total of 527 gig workers were included in the final analysis, of which 80 (15%) reported difficulty finding restrooms while at work, while 447 (85%) did not. Cohort demographic information and characteristics of gig work are presented in Table 1. There were no significant demographic differences between groups. Overall, the study sample was young, female, college‐educated and identified as non‐Hispanic White. Gig workers who reported difficulty finding restrooms more frequently reported food delivery services (42% vs 30%, *p* = 0.03) and ride sharing jobs (25% vs. 14% *p* = 0.03) as primary gig work. Most gig workers (56%) did not work more than 21 h per week, and this was not significantly different between groups (48% vs 43%, *p* = 0.45). There was also no significant difference between groups in terms of duration of gig employment; overall, most gig workers (44%) reported being in their jobs for 24 months or longer.

**Table 1:** Demographic information and characteristics of gig work.

	Difficulty finding restrooms		
	No	Yes	*p* value
N	447 (85%)	80 (15%)	
Age (mean, years)	39.8 (14.3)	37.3 (12.9)	0.142
BMI	28.4 (7.7)	28.8 (8.2)	0.617
Age groups, years			
18−24	55 (12%)	11 (14%)	0.722
25–44	237 (53%)	46 (57%)	
45–64	125 (28%)	20 (25%)	
>= 65	30 (7%)	3 (4%)	
Gender			
Woman	288 (64%)	54 (68%)	0.800
Man	147 (33%)	25 (31%)	
Other, nonbinary, trans	12 (3%)	1 (1%)	
Race/Ethnicity			
White	322 (72%)	47 (59%)	0.089
Black	44 (10%)	14 (18%)	
Asian	20 (4%)	6 (8%)	
Other, Native Amer, or Multi	30 (7%)	8 (10%)	
Hispanic	31 (7%)	5 (6%)	
Education Level			
HS or less	63 (14%)	6 (8%)	0.148
College or more	384 (86%)	74 (92%)	
Locality			
Urban	204 (46%)	32 (40%)	0.257
Suburban	180 (40%)	40 (50%)	
Rural	63 (14%)	8 (10%)	
Health Insurance			
No Insurance	57 (13%)	17 (21%)	0.401
Self‐purchased	85 (19%)	11 (14%)	
From the government	112 (25%)	22 (28%)	
From a partner/spouse	53 (12%)	8 (10%)	
From my employer (not my gig position)	137 (31%)	22 (28%)	
From my gig position	3 (1%)	0 (0%)	
Primary Gig Job			
Ride‐sharing platforms (Uber, Lyft, etc)	64 (14%)	20 (25%)	0.033
Delivery of take‐out food, groceries, goods (DoorDash, Shipt, etc)	134 (30%)	34 (42%)	
Elder and child care services	40 (9%)	3 (4%)	
Pet care (Rover, etc)	43 (10%)	3 (4%)	
Technology testing	10 (2%)	1 (1%)	
Skilled labor platforms (Fiverr, YourMechanic, etc)	34 (8%)	5 (6%)	
Service‐based platforms (TaskRabbit, Handy, etc)	24 (5%)	4 (5%)	
Home care (LawnLove, Handy, etc)	6 (1%)	1 (1%)	
Other	92 (21%)	9 (11%)	
Gig work ≥ 21 h per week	192 (43%)	38 (48%)	0.450
Months at Gig job			
< 6 months	73 (16%)	7 (9%)	0.086
6 or more months	69 (15%)	19 (24%)	
12 or more months	106 (24%)	23 (29%)	
24 or more months	199 (45%)	31 (39%)	
Secondary Gig Job	185 (41%)	41 (51%)	0.101

### 3.1 Toilet Behaviors and Coping Strategies

Gig workers with difficulty finding restrooms reported higher rates of unhealthy toileting behaviors and coping strategies across almost all items queried (Table 2). This included delaying voiding when busy and waiting to use the restroom until at home or until unable to wait any longer. They also more frequently employed coping strategies, such as using UI products while at work, bringing a change of clothes in case of UI, and restricting fluid intake. Gig workers overall most often used restrooms at restaurants, stores, cafés, and rest stops while at work, when not using the restroom at their own home. Those with difficulty finding restrooms were also significantly more worried about public restroom cleanliness (74% vs 54%, *p* = 0.001) and more frequently avoided public restrooms (44% vs 31%, *p* = 0.02). Gig workers with difficulty finding restrooms at work most commonly reported a perception of limited access as the reason for having difficulty finding restrooms (64% vs 28%, *p* < 0.001). On the other hand, reasons such as restroom quality (53% vs. 41%, *p* = 0.07), being busy (68% vs. 69%, *p* = 0.9), embarrassment or privacy (22% vs 15%, *p* = 0.1), and lack of gender options (3% vs. 2%, *p* = 0.6) were not significantly different from those who did not have difficulty finding restrooms.

**Table 2:** Toilet behaviors and coping strategies of gig economy workers.

	Difficulty finding restrooms		
	No	Yes	*p* value
Total N	447 (85%)	80 (15%)	
Restrict fluid intake	39 (9%)	29 (36%)	< 0.001
Bring extra clothes just in case of UI	61 (14%)	19 (24%)	0.020
Need to change clothes for UI	1 (0%)	5 (6%)	< 0.001
Use UI products	36 (8%)	12 (15%)	0.047
Worry about RR cleanliness	243 (54%)	59 (74%)	0.001
Avoid Public RRs	138 (31%)	35 (44%)	0.024
Empty bladder before leaving home	384 (86%)	73 (91%)	0.195
Hold urine until home	81 (18%)	41 (51%)	< 0.001
Delay urinating when busy	177 (40%)	55 (69%)	< 0.001
Wait until stop	194 (43%)	47 (59%)	0.011
Wait until can't hold any longer	71 (16%)	31 (39%)	< 0.001
Wait too long to urinate	51 (11%)	39 (49%)	< 0.001
Location of RR most often used			
Own home	166 (37%)	31 (39%)	0.021
Friends and/or client's home	49 (11%)	1 (1%)	
Restaurants, cafes, convenience stores, and rest stops	193 (43%)	45 (56%)	
Shops, museums, and sites of interest	9 (2%)	1 (1%)	
Other	30 (7%)	2 (2%)	
Limit RR use at work			
Not at all or Occasionally	227 (51%)	17 (21%)	< 0.001
Sometimes	119 (27%)	17 (21%)	
Most or all the time	101 (23%)	46 (57%)	

### 3.2 LUTS and Urinary Conditions

Overall, gig workers reported high rates of urinary symptoms and conditions (Table 3). Those with difficulty finding restrooms reported more frequent and severe LUTS than those with no difficulty, and more often reported a perception of their bladder condition causing them least minor problems (52% vs. 31%, *p* < 0.001). While there were no differences between the groups in recurrent UTI, kidney stones, interstitial cystitis/bladder pain syndrome or mixed UI, significantly more gig workers with difficulty finding the restroom reported OAB, urgency UI, and stress UI, as well as worse symptom severity, measured by the ICIQ‐LUTS questionnaire. More gig workers with difficulty finding restrooms felt their LUTS worsened since starting their gig job (50% vs. 22% *p* < 0.001). On multivariable analysis, self‐reported difficulty finding a restroom while working a gig job was associated with an increased odds of worsening LUTS (OR 3.1, 95% CI 1.8–5.4) adjusting for age, gender, BMI, education level, PPBC, and duration in gig work (Table 4).

**Table 3:** Urinary symptoms and conditions in gig workers.

	Difficulty finding restrooms		
	No	Yes	*p* value
N	447 (85%)	80 (15%)	
Patient Perception of Bladder Condition ( ≥ minor problems)	137 (31%)	42 (52%)	< 0.001
ICIQ LUTS [mean (SD)]			
Total Score	6.8 (5.8)	10.8 (8.1)	< 0.001
Filling symptoms sub score	2.9 (2.3)	4.7 (3.2)	< 0.001
Voiding symptoms sub score	1.6 (1.8)	2.3 (2.4)	0.003
Incontinence symptoms sub score	2.3 (3.4)	3.9 (4.2)	< 0.001
OAB symptom sub score	3.2 (2.6)	5.0 (3.3)	< 0.001
ICIQ‐Urinary Incontinence, Short Form	3.2 (4.7)	5.4 (6.1)	< 0.001
Recurrent UTI	19 (4%)	2 (2%)	0.461
Kidney Stones	27 (6%)	4 (5%)	0.716
Bladder Pain Syndrome	7 (2%)	2 (2%)	0.553
OAB	144 (32%)	45 (56%)	< 0.001
Urgency	119 (27%)	42 (52%)	< 0.001
Urgency UI	72 (16%)	23 (29%)	0.007
Stress UI	63 (14%)	20 (25%)	0.014
Mixed UI	43 (10%)	13 (16%)	0.076
Change in LUTS since starting Gig job			
Worse	100 (22%)	40 (50%)	< 0.001
No change	320 (72%)	34 (42%)	
Better	27 (6%)	6 (8%)	

**Table 4:** Results of multivariable logistic regression for associations with difficulty finding a restroom while at Gig job.

	Odds Ratio	95% CI	*p* value
LUTS change with Gig job			
Worse	3.1	[1.8,5.4]	< 0.001
No change	ref		
Better	1.9	[0.7,5.0]	0.217
Age (years)	1.0	[1.0,1.0]	0.143
Gender			
Woman	ref		
Man	1.0	[0.6,1.7]	0.898
Other, nonbinary, trans	0.4	[0.0,3.5]	0.415
BMI	1.0	[1.0,1.0]	0.863
Months at Gig job			
< 6 months	ref		
6 – 12 months	3.2	[1.2,8.4]	0.018
12 – 24 months	2.1	[0.8,5.3]	0.118
≥ 24 months	1.8	[0.7,4.3]	0.222
Education Level			
High school or less	ref		
College or more	1.5	[0.6,3.8]	0.372
Patient Perception of Bladder Condition			
None or very minor problems	ref		
≥ Minor problems	1.9	[1.1,3.3]	0.020

## 4 Discussion

This study is the first to examine urinary symptoms and bladder dysfunction specifically within gig economy employees. Overall, these workers reported high prevalence of LUTS and unhealthy toileting behaviors, which may have long‐term impacts on overall bladder health. Consistent with our hypothesis, those who reported difficulty finding restrooms while working in their gig job had greater severity of LUTS and demonstrated higher rates of unhealthy toileting behaviors.

Few studies have examined health aspects of gig workers, which is an increasing proportion of the U.S. population. Gig workers are especially vulnerable in three ways: occupational vulnerabilities, precarity, and platform‐based vulnerabilities [16]. This study raises awareness to occupational and platform‐based vulnerability associated with limited toilet access while working. The association between toileting behaviors, LUTS, and overall bladder health is complex, and understanding how social and environmental factors, such as those related to occupation, impact LUTS development, quality of life, and economic burden of disease can help guide patient counseling and subsequent behavior‐based treatment.

In our study, workers in gig jobs revolving around transport (i.e. food delivery or ride share jobs) more often reported difficulty finding restrooms, as expected given the lack of reliable restroom access while traveling. While there is limited data on gig workers, there are data addressing similar occupations with limited toilet access, such as taxi drivers and operating room staff. Multiple studies demonstrate the correlation between taxi drivers and genitourinary pathology, such as chronic prostatitis, infertility, and bladder cancer [12]. Limited access to restrooms on the job has also been linked to common adoption of coping behaviors. Healthcare professionals working in the operating room, where toilet access and breaks are limited, were at the highest risk of genitourinary pathologies as compared to those who were not in the operating room, and used decreased fluid intake as a coping mechanism [26]. Likewise, prior literature on coping behaviors utilized by women with limited access to toilets in occupational settings reported delayed urination and limited fluid intake as common strategies [27]. The present study found gig workers likewise had a high prevalence of genitourinary pathology, as well as significant use of coping bladder behaviors and strategies. In addition to challenges regarding time and physical access to restroom spaces, a large portion of gig workers reported avoiding public restrooms when available due to concerns regarding cleanliness. Sanitation has been previously recorded as a major factor leading adults to avoid or delay restroom use [3]. Our findings confirm the multifactorial aspect of toileting behavior with voiding habits among gig workers being impacted by location, time, and personal restraints, which may contribute to the development of LUTS in these patients.

Findings in the present study are consistent with prior studies linking increased rates of urinary symptoms to occupations with inherent limitations or restrictions in restroom access and practices of delayed voiding [5, 6, 7, 8, 9, 10]. While other studies looking only at women who limit restroom use at work have shown high rates of UI, our study of both male and female gig workers showed higher rates of OAB symptoms in those with limited restroom use [3]. A previous study of mixed‐gender taxi drivers showed 10% of drivers reported OAB and 29% reported moderate to severe LUTS, which is similar to our rates in gig workers without difficulty finding restroom use, but lower than those with difficulty finding restrooms [12]. This may suggest a change in restroom access or perception of restroom access for gig workers. Prior studies have not explored the impact of duration of employment on LUTS, however in this study those with difficulty finding restrooms reported worsening of their LUTS since starting their gig work, especially in the first 6 to 12 months regardless of age, gender, type of primary gig work, duration within the gig economy, or time spent within gig job per work. Our findings suggest that gig work with limited restroom access may play a role in developing or exacerbating bladder symptoms. Alternatively, gig workers with LUTS may find more limitations in restroom access during gig work. Either way, there appears to be a negative association between restroom access and bladder symptoms among our subset of gig workers. While our study was not designed to assess risk of urinary conditions over time, in the future it would be important to study the potential long‐term implications for bladder health or urinary conditions after working in a gig job for a period of time.

Based on the limited data on gig employment in the current literature, it is clear that additional research is needed. Especially in light of the recent COVID19 pandemic, the gig economy has already exponentially expanded and will likely continue to grow. As a result, the unhealthy adaptative mechanism and toileting behaviors noted in this study may become more prevalent, and the long‐term impacts on overall bladder and mental health remain unknown [28]. Therefore, additional research exploring the impacts of work within the gig economy, ease of restroom finding, and bladder health is imperative to help create solutions that reduce the burden of healthcare costs and increase quality of life [28]. Moreover, understanding the patient population that makes up this workforce, their ability to access healthcare, and the health impact of this occupation will allow providers and legislators to advocate for this ever‐growing population. Lastly, information distributed from studies such as this one can help raise bladder health awareness among gig workers, which may in turn encourage protective behaviors to promote bladder health and options for bathroom access while working. Some cities provide taxi relief stands for drivers, where taxi drivers can park and leave their vehicle for up to a 1 h break, providing an opportunity for drivers to use the restroom [29]. We believe similar systems could be advocated for gig workers within various platforms.

There are several limitations to consider in this study. Our survey is subject to recall bias since we relied on participants self‐reported symptoms. People may overestimate time spent doing an activity, such as number of hours worked. Furthermore, we are not aware of any validated tool to evaluate bladder symptoms in the mixed gender gig population. The validated questionnaires used in this study were developed and validated only in women, whereas our study surveyed both men and women using these tools. As with any survey, our survey was likely subject to selection bias, as workers with bladder symptoms are more likely to respond to our initial recruitment email and the electronic nature of the survey limits access to those with electronic access. However, given the majority of gig jobs occur electronically, it is believed that an electronic survey likely appealed to our target population. Additionally, due to the cross‐sectional nature of our study, we are unable to determine causality between LUTS and gig work. We also are unable to compare between a sample of participants not in gig work, as we did not include this in our initial study design, but this would be an interesting area of study for the future. Our sample size is limited due to targeting a specific population of workers within a large heterogeneous database and a power calculation could not be accurately performed a‐priori as there is not a similar population for comparison. As our results are limited by participants available in ResearchMatch, they are not generalizable to the general population in the U.S. Our respondents were less diverse than the general U.S. population, more likely to identify as female, and more likely to have completed high school or higher education [30].

Despite these limitations, this study provides further insight on a rising employment platform as a social driver of bladder health. Gig workers are one of many populations at risk to develop urinary issues due to occupational hazards; therefore it is important for clinicians to consider the social history of their patients and the challenges they might face in toileting due to their job. Toileting habits in this population should be identified early so that timely interventions promoting healthy toileting behaviors can be provided to those at risk. Additionally, advocacy for greater workplace protections and benefits, such as restroom breaks and access to cleanly toilet facilities, could greatly benefit this population.

## 5 Conclusions

Bladder behaviors of gig workers are affected by their work within the gig economy and ease of restroom access while at work. This subset of gig workers overall demonstrate unhealthy toileting behaviors and coping strategies while at work; however those with difficulties in accessing restrooms while working demonstrate such behavior and strategies at higher rates. Limitations in reliable restroom access for gig workers may contribute to poor toileting habits which may further exacerbate underlying bladder conditions. It is important to identify at‐risk groups, assess their voiding habits, and educate them on healthy toileting behaviors to help prevent and manage bladder issues. Further research expanding our knowledge of bladder health and employment in the gig economy is needed to better understand this unique population.

## Author Contributions

All authors contributed to the creation of this study and manuscript.

## Ethics Statement

This study was approved by Institutional Review Board of Vanderbilt University Medical Center (IRB #190528).

## Consent

Complete written informed consent was obtained from participants for the publication of this study.

## Permission to Reproduce Material From Other Sources

There is no material from other sources in manuscript to provide permission to reproduce.

## Conflicts of Interest

The authors declare no conflicts of interest.

## Data Availability

The data that support the findings of this study are available from the corresponding author upon reasonable request. The data that support the findings of this study are available upon request.
